# Empirical relationships between tree fall and landscape-level amounts of logging and fire

**DOI:** 10.1371/journal.pone.0193132

**Published:** 2018-02-23

**Authors:** David B. Lindenmayer, Wade Blanchard, David Blair, Lachlan McBurney, John Stein, Sam C. Banks

**Affiliations:** 1 Fenner School of Environment and Society, The Australian National University, Canberra, Australian Capital Territory, Australia; 2 National Environmental Science Programme, Threated Species Recovery Hub, Fenner School of Environment and Society, The Australian National University, Canberra, Australian Capital Territory, Australia; 3 Long Term Ecological Research Network, Fenner School of Environment and Society, The Australian National University, Canberra, Australian Capital Territory, Australia; Chinese Academy of Forestry, CHINA

## Abstract

Large old trees are critically important keystone structures in forest ecosystems globally. Populations of these trees are also in rapid decline in many forest ecosystems, making it important to quantify the factors that influence their dynamics at different spatial scales. Large old trees often occur in forest landscapes also subject to fire and logging. However, the effects on the risk of collapse of large old trees of the amount of logging and fire in the surrounding landscape are not well understood. Using an 18-year study in the Mountain Ash (*Eucalyptus regnans*) forests of the Central Highlands of Victoria, we quantify relationships between the probability of collapse of large old hollow-bearing trees at a site and the amount of logging and the amount of fire in the surrounding landscape. We found the probability of collapse increased with an increasing amount of logged forest in the surrounding landscape. It also increased with a greater amount of burned area in the surrounding landscape, particularly for trees in highly advanced stages of decay. The most likely explanation for elevated tree fall with an increasing amount of logged or burned areas in the surrounding landscape is change in wind movement patterns associated with cutblocks or burned areas. Previous studies show that large old hollow-bearing trees are already at high risk of collapse in our study area. New analyses presented here indicate that additional logging operations in the surrounding landscape will further elevate that risk. Current logging prescriptions require the protection of large old hollow-bearing trees on cutblocks. We suggest that efforts to reduce the probability of collapse of large old hollow-bearing trees on unlogged sites will demand careful landscape planning to limit the amount of timber harvesting in the surrounding landscape.

## Introduction

Large old trees are critical elements of stand structural complexity (*sensu* [1]) in forests worldwide [2–4]. They play an array of key ecological roles ranging from storing disproportionately large amounts of carbon to acting as sources of flowers, pollen and seeds, and providing habitat for numerous elements of the biota (reviewed by [3]).

Given that large old hollow-bearing trees they are keystone structures (*sensu* [5,6]) in many forest ecosystems, it is critical to quantify and better understand the factors influencing where they occur and why they occur where they do. As in the case of all long-lived organisms, adult mortality, including tree collapse, is a key phase in the dynamics of populations of large old trees [3]. In the case of such trees, the survival of individual living organisms is important but the primary ecological relevance is the survival of the keystone ecological structure itself (which may be a living or dead tree). Therefore, documenting the factors affecting the collapse of large old trees is essential to understanding the distribution, abundance and dynamics of current populations and predicting future populations of these trees.

Two broad categories of factors that influence the dynamics of large old trees are human disturbances such as logging [7,8] and natural disturbances like wildfire [9]; their effects at a tree and stand level have been well documented in a large number of studies. In the case of logging, large old trees may be directly removed by harvesting or those that are retained on cutblocks may collapse soon after logging operations have been completed [10]. Similarly, fire may directly consume large old trees on burned sites. Both logging and fire can be spatially heterogeneous [11] leaving mosaics (*sensu* [12]) of logged and unlogged areas or, similarly, unburned areas interspersed with burned patches [13,14]. The survival of large old trees in forest stands persisting in these mosaic landscapes shaped by natural and human disturbance is not well understood. It is therefore important to determine whether there are landscape context effects (*sensu* [15]) in which tree fall on sites is affected by the amount and/or the spatial pattern of disturbance in the surrounding landscape. Globally, forest landscapes are subject to increasing amounts of natural disturbance [16] and also human disturbance [17] and it is therefore critical to understand their landscape-level effects on large old trees. This is useful because many forest management prescriptions require retention of unlogged stands within the landscape, such as variable retention harvesting strategies for the future recruitment of hollow-bearing trees, protection of existing critical habitat elements, and retention of sensitive areas such as riparian zones. In fire-affected forests, unburnt ‘refuges’ are important for biodiversity [18,19] and often persist in the context of fire in the surrounding landscape [20]. Our study addresses whether the persistence of large old trees in such stands is itself affected by the amount of disturbed forest in the surrounding landscape.

We explored evidence for landscape context effects on the collapse of large old trees in the Mountain Ash (*Eucalyptus regnans*) ecosystem in the Central Highlands of Victoria, south-eastern Australia. This ecosystem has been subject to extensive and intensive wildfires in the past decade [21], and to widespread clearcut logging [22,23]. There are also landscapes where there has been limited or no logging or fire, making the Mountain Ash ecosystem a valuable one in which to quantify the associations between the collapse of large old trees and landscape-scale patterns of forest cover. Moreover, the mortality and collapse of large old trees in Mountain Ash forests has underpinned the classification of the ecosystem as Critically Endangered under the IUCN Red Listed Ecosystem criterion [24]. This underscores the importance of understanding the factors contributing to tree collapse in these ecosystems.

Specifically, we addressed two key questions in this study.

1. What are the effects of the amount of logging and fire in the surrounding landscape on the probability collapse of large old hollow-bearing trees? At the outset of this investigation, we postulated there would be a positive association between the probability of collapse of large old trees on sites and the amount of logging in the surrounding landscape. This was based on other studies which have highlighted how altered patterns of forest cover associated with forest clearing and logging operations alter climatic conditions such as air flow and windiness (e.g. [25–27]) leading to accelerated rates of tree fall [28–31] including the collapse of large old trees [32]. We also postulated that the probability of collapse of large old trees on sites would be positively associated with the amount of the surrounding landscape that was burned. This prediction was also based on altered microclimatic conditions, particularly wind speed and wind fetch, which can occur when the patterns of forest cover are altered by wildfire (e.g. [13,33,34]).
2. Is there an interaction between the probability of collapse of large old hollow-bearing trees and the amount of logging and the amount of fire in the surrounding landscape? Such an interaction, if it exists, might mean, for example, that the collapse of large old trees associated with logging in the surrounding landscape is greater when those landscapes have been burned. On this basis, we forecast higher levels of collapse in burned landscapes also subject to logging than in landscapes where only logging occurred or where only fire had occurred but not both. This prediction was based on the premise that altered patterns of air movement associated with creating clearcuts might exacerbate the probability of tree collapse in other parts of the same landscape which had been burned.

## Methods

### Study area and surveys of large old trees

**Fig 1:**
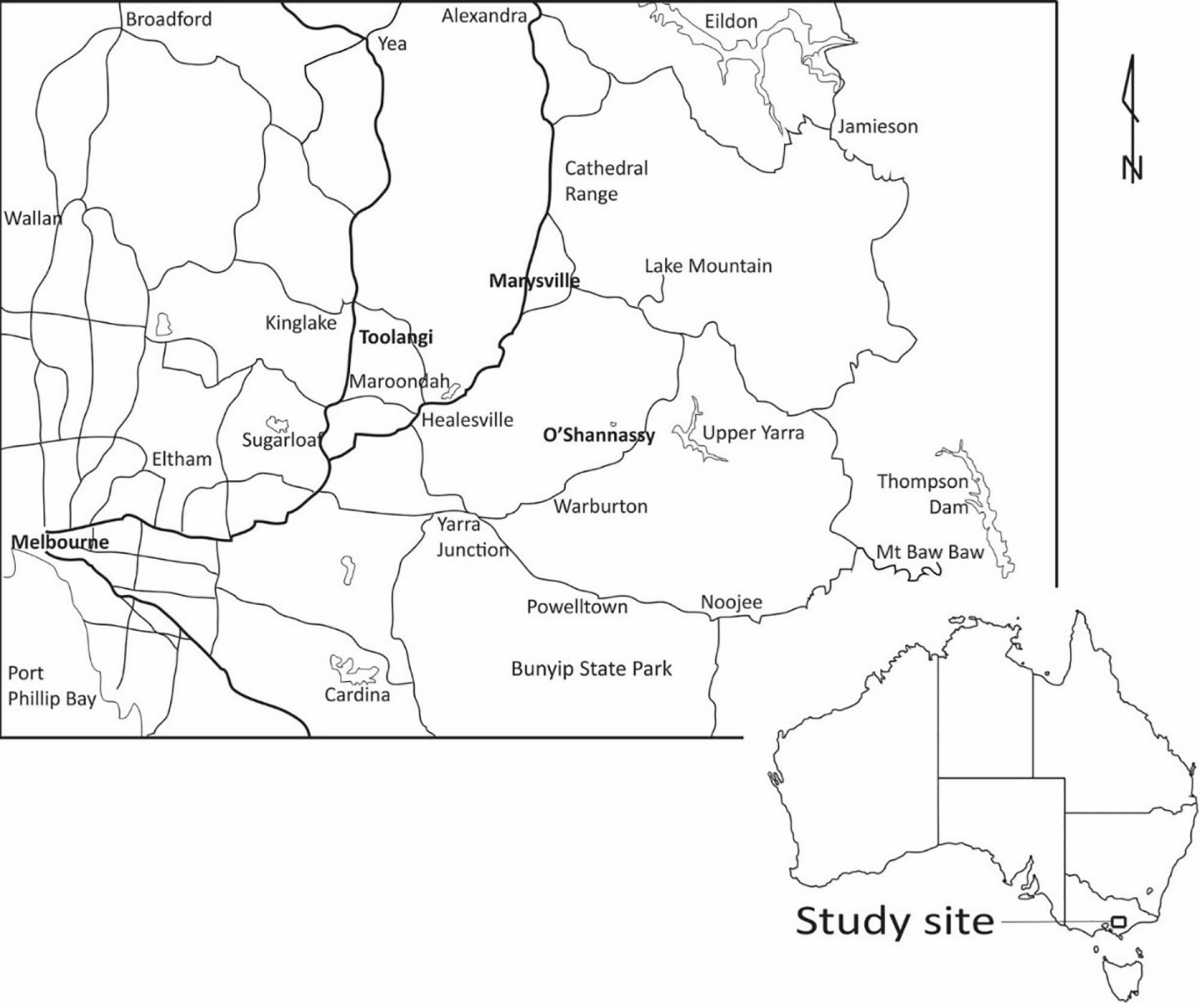
The location of the Central Highlands region of south-eastern Australia where studies of the abundance and transitions of large old hollow-bearing trees have been conducted.

We completed this study in the Mountain Ash (*Eucalyptus regnans*) of the Central Highlands of Victoria, south-eastern Australia (Fig 1). There is approximately 157 000 ha of Mountain Ash forest in the Central Highlands. The primary form of natural disturbance in this forest is high-severity, stand-replacing or partial stand-replacing wildfire; the last major conflagration was in 2009 when 78 300 ha was burned [35]. Approximately 80% of the Mountain Ash forest estate is located in areas broadly designated for wood production and the predominant silvicultural system is clearcutting in which cutblocks of 15–40 ha are harvested [22].

We established 104 long-term ecological research sites in the Mountain Ash forest in the Central Highlands of Victoria. Each site was 1 ha in size, on which we completed repeated measurements of the number and condition of large old hollow-bearing trees over an 18-year period between 1997 and 2015. We marked all hollow-bearing trees with permanent metal tags and unique identifying numbers to facilitate re-measurement. Notably, none of our 104 long-term sites was subject to logging, although immediately adjacent or surrounding areas were subject to timber harvesting between 1997 and 2015 at approximately 30% of our sites.

For the purposes of this study, we defined a hollow-bearing tree as any tree (live or dead) measuring > 0.5 m dbh and containing an obvious cavity as determined from careful visual inspection using a pair of binoculars (hollows in trees smaller than 0.5 m dbh rarely occur and are not suitable for hollow-dependent mammals or birds in this landscape). We classified all hollow-bearing trees on all long-term sites into one of nine forms based on the condition and level of decay. These are: Form 1: Ecologically mature, living tree; Form 2: Mature living trees with a dead or broken top; Form 3: Dead tree with most branches still intact; Form 4: Dead tree with 0–25% of the top broken off; branches remaining as stubs only; Form 5: Dead tree with top 25–50% broken away; Form 6: Dead tree with top 50–75% broken away; Form 7: Solid dead tree with 75% of the top broken away; Form 8: Hollow stump. We added a ninth category–Form 9: Collapsed tree–in the later years of our surveys. (See [36])

### Ethics statement

Our research required no ethics approvals as we were undertaking non-destructive vegetation sampling without collection of any vegetation samples and we were not studying fauna. Our sites were all in publicly accessible locations on public land controlled by the Victorian Government, and therefore no special permission was required.

### Covariates for use in statistical modelling

Our key response variable was the probability of collapse of a large old hollow-bearing trees tree. At the tree level, a key covariate was tree form corresponding to the decay status of the tree. To facilitate our analysis, we grouped tree forms into three categories: living trees (forms 1 and 2 combined), moderately decayed dead trees (forms 3–5 combined), and highly decayed trees (forms 6–8 combined).

At the landscape level, we calculated the proportion of 20 m x 20 m pixels logged between 1997 and 2015 in a 2 km radius circle around the centroid of each long-term site. We also calculated the proportion of 20m x 20 m pixels burned (at any level of severity) in the 2009 fire in a 2 km radius circle around the centroid of each long-term site. The buffer size of 2 km was based on earlier work [37] suggesting that changes in microclimatic conditions associated with disturbances such as clearfell logging might occur over distances of 1–2 km. The 2009 fire was the only major conflagration that occurred during our 18-year investigation.

As a measure of the spatial pattern of timber harvesting, we calculated the median logarithmic distance between harvested cutblocks. We assigned a log distance of zero to sites with no logging in the surrounding landscape. We also calculated a measure of distance from fire and logging disturbed pixels to the centroid of our sites. We also explored a wide range of other spatial metrics drawn from the program FRAGSTATS [38]. Finally, we constructed a categorical variable corresponding to whether a given site was located with closed water catchments where logging operations are largely excluded or whether it was in wood production forest where clearcutting operations take place.

### Statistical analysis

To answer the two questions posed at the outset of this study (see Introduction), we used multi-level Bayesian binary logistic regression with collapse status as the response variable, the above mentioned covariates as potential predictor variables, and site as the grouping variable. Follow preliminary analysis, we excluded catchment and median log distance from subsequent modelling as there was strong multi-colinearity with the other variables (correlations > 0.5 in absolute value). We also found that measure of distance from fire and logging disturbed pixels to the centroid of our sites were highly correlated with other spatial pattern variables and excluded them from our analyses. We looked for two-way interactions between the amount of logging, fire and tree form and also investigated quadratic effects for the amount of timber harvesting and fire to check for potential non-linear relationships. We used non informative priors on all model parameters and model selection was done using LOOIC [39]. In interest of model parsimony, we selected the simplest model within two LOOIC units of the minimum LOOIC model. We constructed models using the brms (Bayesian Regression Models using Stan) package ([40] in R [41]). We standardized the continuous variables, harvest and fire, to have a zero mean and standard deviation of 1 prior to analysis.

## Results

We recorded 737 hollow-bearing Mountain Ash trees on our 104 field sites. The number of hollow-bearing trees per site ranged from 1 to 23 with a mean of 7.1 per site. Our initial sample of trees in 1997 contained no collapsed trees (Form 9) (Table 1). By 2015, 41% of large old hollow-bearing trees standing in 1997 had collapsed.

**Table 1:** The number of standing hollow-bearing trees in 1997 and collapsed hollow-bearing trees in 2015.

Tree form	No. of hollow-bearing trees in 1997	Collapsed hollow-bearing trees in 2015 as percentage of 1997 number (2015 number in parentheses)
1	161	7.4% (12)
2	31	19.4%(6)
3	91	31.9%(29)
4	66	43.9%(29)
5	77	55.8%(43)
6	124	72.6%(90)
7	156	69.9%(109)
8	31	71.0% (22)

### Statistical model of relationships between the probability of collapse of large old trees and landscape-level disturbance

We tested for both linear and quadratic effects for the various covariates but found no evidence for non-linear effects. We focus exclusively on linear effects in the remainder of this paper.

**Fig 2:**
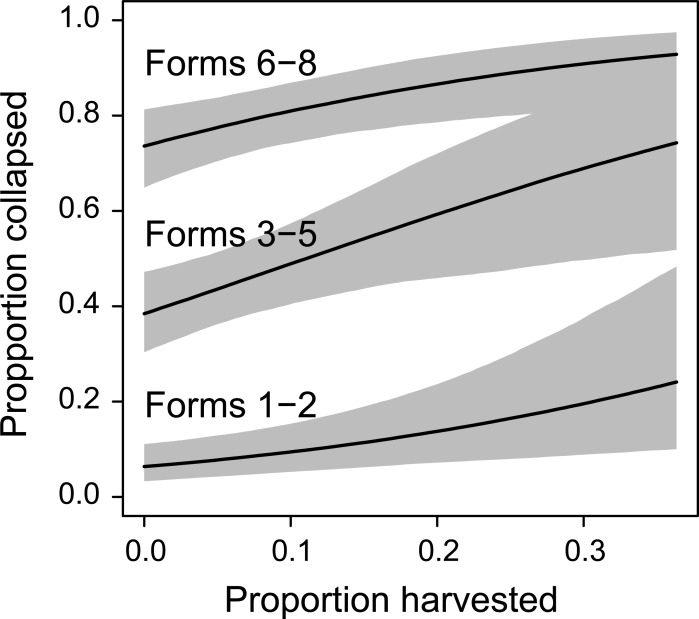
Relationships between the probability of collapse of hollow-bearing trees between 1997 and 2015 and the amount of logging in the surrounding landscape. The three components of the relationship show patterns for trees in forms 1 and 2 (category 1), forms 3–5 (category 2) and forms 6–8 (category 3). The amount of fire is held at the mean level in the landscape.

**Fig 3:**
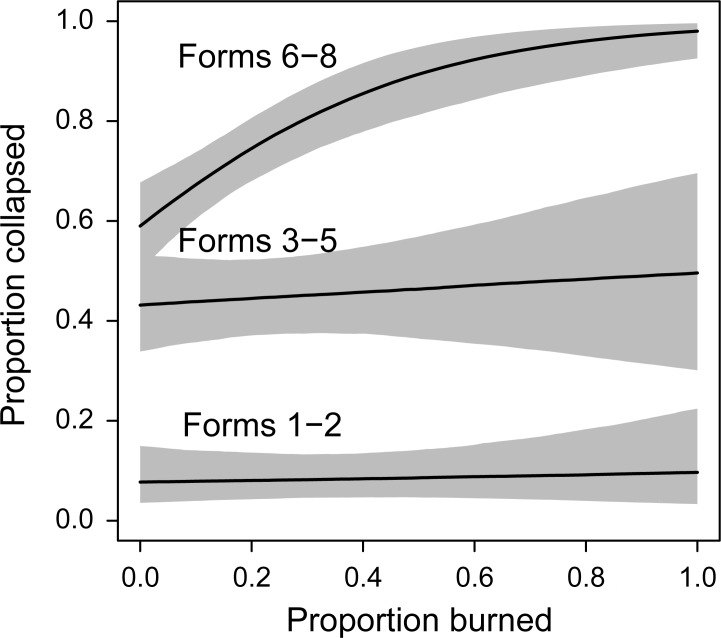
Interaction between the probability of collapse of different forms of hollow-bearing trees between 1997 and 2015 and the amount of fire in the surrounding landscape. The probability of collapse of trees in forms 6–8 increased with increasing amount of burned area in the surrounding landscape, whereas the probability of collapse of other tree form categories remained relatively unchanged with the increasing amounts of fire. The amount of harvesting is set at the mean value in the landscape.

We found the best fitting model for the probability of tree collapse between 1997 and 2015 contained evidence of a positive relationship with the amount of logged forest in the surrounding 2000 m radius polygon (S1 Table and S2 Table). That is, the probability of collapse increased with an increasing amount of logged forest in the surrounding landscape (Fig 2). We also found evidence for an interaction between tree form and the amount of fire in the surrounding 2000 m polygon (Fig 3). The probability of collapse of trees in forms 6–8 increased with increasing amount of burned area in the surrounding landscape, whereas the probability of collapse of other tree form categories remained relatively unchanged with the increasing amounts of fire (Fig 3).

## Discussion

Understanding the effects of landscape-level disturbance on keystone structures such as large old hollow-bearing trees is a critical part of forest management given that the amount of natural and human disturbance of forest landscapes is increasing [16] and populations of large of trees are declining in many forest ecosystems globally [3,42].

### What are the effects of the amount of logging and fire on the probability collapse of large old hollow-bearing trees?

We found that the probability of tree collapse increased with increasing amounts of cut forest in the surrounding landscape (Fig 2). We suggest the likely reason for this finding is changes in wind movement patterns through forested landscapes when trees are either removed by logging or when they are burned. Altered wind speeds and wind fetch associated with logging operations have been documented in numerous forest ecosystems around the world (e.g. [25,27–30,43], including in the Mountain Ash forests that were the focus of this investigation [36].

Our analyses revealed a positive relationships between the probability of collapse of large old hollow-bearing trees and the amount of burned forest in the surrounding landscape (Fig 3). Relative to the effects of logging on microclimatic conditions in adjacent areas, to the best of our collective knowledge, substantially less work has been done on inter-relationships between tree fall and changes in microclimate associated with the effects of landscape-level high severity fire [13,34]. The size of open areas created by tree loss following high-severity fire will typically be far smaller than those associated with logging operations like clearcutting with a single cutblocks in Mountain Ash forest (usually 15–40 ha; [44]) but nevertheless fire-derived change in landscape levels of forest cover can influence key processes like the collapse of large old hollow-bearing trees. An important outcome of our analysis was tree fall was affected by the composition of the landscape with 2 km of a site. This result is broadly consistent with other studies showing long distance influences of landscape change on forest tree dynamics [45,46], including the widespread depletion of carbon stocks close to forest edges [47].

### Potential effects of the spatial pattern of logging and fire on the probability of collapse of large old hollow-bearing trees?

We found an effect of the amount of habitat modification in the surrounding landscape on tree fall, but does the spatial pattern of logging and fire matter? There has been considerable discussion in the ecological literature concerning the relative importance for biodiversity of the amount of habitat versus the spatial configuration of that habitat (e.g. [48,49]). Notably, there is a general paucity of work on the reverse effect, the loss of habitat (in this case the loss of large old trees) resulting from different spatial patterns of logging (and also fire) in the surrounding landscape. Other earlier studies, including those based on simulation modeling, have suggested that the spatial pattern of disturbance can have significant effects on biodiversity and key ecosystem processes (e.g. [11]). We were unable to quantify the relative effect of the spatial pattern versus amount of logging on tree fall in the present study. This was because of substantial confounding between the amount and spatial pattern of both logging and of fire in the landscape, preventing their conjoint inclusion in the statistical models we constructed. That is, for example, the more logging that occurred in the landscape, the closer the distance between cutblocks. Indeed, in the preliminary stages of this investigation we trialed a wide range of spatial metrics in an effort to overcome multi-colinearity problems, including through the use of various measures in computer packages such as FRAGSTATS [37,50]. The solution to this problem of a lack of independence in the amount and spatial pattern of disturbance is a major challenge to resolve (and may be unresolvable) given the inherent lack of ability to control where and when in the landscape, disturbances such as large-scale, unplanned, high-severity wildfires occur.

### Is there an interaction between the probability of collapse of large old hollow-bearing trees and the amount and/or spatial pattern of logging and the amount and/or spatial pattern of fire in the surrounding landscape?

We found no evidence of an interaction between the amount of logging and the amount of burned forest on the probability of collapse of large old hollow-bearing trees. The paucity of such effects was contrary to our prediction at the outset of this investigation (see Q2 in the Introduction), especially as logging and fire interact in other ways such as the risk of elevated high severity crown fire within stands regenerating after timber harvesting [51]. The reasons for this finding remain unclear. Our results did, however, contain evidence of an interaction between tree form and the amount of fire in the surrounding landscape. Highly decayed hollow-bearing trees (forms 6–8) on sites where there had been a large amount of fire in the surrounding landscape were more likely to collapse than trees on sites where only a limited amount of the surrounding forest had been burned (Fig 3). We suggest that there is likely to be two interacting factors underpinning this result. First, patterns of spatial contagion in the 2009 wildfire mean that landscapes where there has been extensive fire and are likely to support sites that are also likely to have been burned, thereby damaging hollow-bearing trees in such areas. Second, and related to the first explanation, is that trees in forms 6–8 are the most decayed kinds of hollow-bearing trees and are characterized by large amounts of flammable dead wood, making them more prone to collapse in the event of them being burned (Fig 3).

### Management implications

The maintenance of populations of large old hollow-bearing trees is critical in many ecosystems globally (reviewed by [3,4]). The maintenance of large old hollow-bearing trees is likewise essential in Mountain Ash ecosystems for a wide range of reasons including long-term carbon storage [52] and the persistence of populations of cavity-dependent vertebrates such as several species of arboreal marsupials of conservation concern [53]. Indeed, the rapid and ecosystem-wide decline in populations of large old hollow-bearing trees is one of the key reasons why Mountain Ash ecosystems were classified as Critically Endangered under the formal IUCN Red Listed Ecosystem protocol [24].

Previous work in Mountain Ash forests has indicated that a range of site-level and tree-level factors can influence the probability of collapse of large old hollow-bearing trees [9,54]. The new work presented in this paper has indicated that landscape-level factors are also important, with the amount of logging up to 2 km away affecting the risk of tree collapse. Large old hollow-bearing trees are already at high risk of collapse in our study area [24] and the empirical analyses presented in this investigation indicate that the addition of logging operations in the surrounding landscape will further elevate that risk. Current logging prescriptions require the protection of large old hollow-bearing trees on cutblocks, although the effectiveness of such actions is often limited [44]. On unlogged sites, we suggest that efforts to reduce the probability of collapse of large old hollow-bearing trees requires careful landscape planning to limit the amount of timber harvesting in the surrounding landscape. Such planning will be particularly important around areas known to presently support large numbers of such trees. These include patches of old growth forest and parts of landscapes dominated by areas of steep, east-facing and west-facing slope [8]. Buffers of unlogged forest also may be required to better protect places with high concentrations of such trees, with our data indicating they may need to be at least 2 km in radius to reduce the risk of tree collapse.

In summary, we have demonstrated that changes in landscape-level forest cover resulting from human and natural disturbance can be strongly associated with the loss of keystone structures in forest ecosystems. The key ecological process of the collapse of large old trees manifests at an individual tree scale but is influenced by factors occurring at much larger scales. This, in turn, underscores the importance of implementing conservation strategies that account for such scale effects, including those at landscape scales that can influence dynamic processes at small scales.

## Supporting information

S1 Table List of models ranked by leave one out cross-validation information criteria. (DOCX)

S2 Table Model coefficients and 95% credible intervals for the best fitting model. (DOCX)
