# Supplementary material for: Empirical relationships between tree fall and landscape-level amounts of logging and fire
Source: PLoS One. 2018 Feb 23;13(2):e0193132. doi: 10.1371/journal.pone.0193132 (PMC5825053; doi:10.1371/journal.pone.0193132)
Supplement: S1 Table — (DOCX) [file pone.0193132.s001.docx]

**Supporting Information**

**S1 Table:** **Leave One Out Cross-validation Information Criteria (LOOIC) for each of the models considered.** The best fitting model is indicated in bold.

| **Model** | **LOOIC** |
| --- | --- |
| Harvest | 937.15 |
| Fire | 938.88 |
| Form | 806.63 |
| Harvest + Fire + Form | 798.36 |
| Fire + Form | 802.85 |
| Harvest + From | 803.33 |
| Harvest + Fire | 927.23 |
| Harvest + Fire + Form + Harvest x Form | 810.06 |
| Harvest + Fire + Form + Fire x Form | **788.68** |
| Harvest + Fire + Form + Harvest x Fire | 798.54 |
| Harvest + Fire + Form + Fire x Form + Harvest^2 | 790.43 |
| Harvest + Fire + Form + Fire x Form + Fire^2 | 790.11 |
