# Supplementary material for: Empirical relationships between tree fall and landscape-level amounts of logging and fire
Source: PLoS One. 2018 Feb 23;13(2):e0193132. doi: 10.1371/journal.pone.0193132 (PMC5825053; doi:10.1371/journal.pone.0193132)
Supplement: S2 Table — (DOCX) [file pone.0193132.s002.docx]

**Supporting Information**

**S2 Table:** **Model coefficients and 95% credible intervals for the best fitting model.** Comparisons of the intercepts and slopes for amount of fire in the surrounding 2km in the landscape among the three tree forms (1-2, 3-5, 6-8; see [1]) are also presented. Note that the coefficients are on the logit-scale and the continuous variables are reported on the standardized scale (i.e. they have been scaled to have zero mean and standard deviation 1).

| **Coefficient** | **Posterior Mean** | **Lower 95% CI** | **Upper 95% CI** |
| --- | --- | --- | --- |
| Form 1-2 Intercept | -2.43 | -3.06 | -1.87 |
| Form 3-5 Intercept | -0.20 | -0.51 | 0.11 |
| Form 6-8 Intercept | 1.29 | 0.92 | 1.70 |
| Harvest | 0.31 | 0.09 | 0.55 |
| Form 1-2 Fire | 0.08 | -0.38 | 0.55 |
| Form 3-5 Fire | 0.08 | -0.25 | 0.42 |
| Form 6-8 Fire | 1.16 | 0.65 | 1.75 |
| Group level SD | 0.55 | 0.14 | 0.89 |
| **Comparison of Intercepts** | |  |  |
| Form 3-5 vs 1-2 | 2.23 | 1.58 | 2.90 |
| Form 6-8 vs 1-2 | 3.73 | 3.05 | 4.47 |
| Form 6-8 vs 1-2 | 1.50 | 1.05 | 1.96 |
| **Comparison of Fire Slopes** | |  |  |
| Form 3-5 vs 1-2 | 0.01 | -0.54 | 0.53 |
| Form 6-8 vs 1-2 | 1.09 | 0.40 | 1.81 |
| Form 6-8 vs 1-2 | 1.08 | 0.47 | 1.74 |

**Reference**

1. Lindenmayer, D.B., Blanchard, W., Blair, D., McBurney, L. and Banks, S. Environmental and human drivers of large old tree abundance in Australian wet forests. Forest Ecology and Management. 2016;372: 226-235.
